# Tazarotene-Induced Gene 3 (TIG3) Induces Apoptosis in Melanoma Cells Through the Modulation of Inhibitors of Apoptosis Proteins

**DOI:** 10.3390/biomedicines13071749

**Published:** 2025-07-17

**Authors:** Chun-Hua Wang, Lu-Kai Wang, Fu-Ming Tsai

**Affiliations:** 1 Department of Dermatology, Taipei Tzu Chi Hospital, Buddhist Tzu Chi Medical Foundation, New Taipei City 231, Taiwan; dermawang@gmail.com; 2 School of Medicine, Tzu Chi University, Hualien 970, Taiwan; 3 National Center for Biomodels, National Institutes of Applied Research, Taipei 115, Taiwan; 2407026@niar.org.tw; 4 Department of Research, Taipei Tzu Chi Hospital, Buddhist Tzu Chi Medical Foundation, New Taipei City 231, Taiwan

**Keywords:** tazarotene-induced gene 3, retinoid-induced gene 1, melanoma, apoptosis, cIAP-1, Livin, HTRA2

## Abstract

**Background/Objectives:** Retinoic acid has been shown to inhibit melanoma progression; however, its underlying mechanisms remain unclear. In this study, we investigated the role of the retinoic acid-inducible gene TIG3 in regulating melanoma cell growth, as well as elucidating its involvement in apoptosis. **Methods:** The expression of TIG3 in melanoma tissues was analyzed using a cDNA microarray. Cell viability and cell death were measured using the WST-1 and LDH assay kits, respectively. The gene expression changes that were induced by TIG3 were identified through RNA sequencing, while apoptosis-related pathways were examined using a human apoptosis protein array. The protein expression levels were further validated using Western blot analysis. **Results:** TIG3 expression was significantly downregulated in melanoma tissues. The overexpression of TIG3 in melanoma cells led to reduced cell viability and increased cell death. TIG3 suppressed the expression of several apoptosis-regulating proteins, including PON2, Fas, cIAP-1, Claspin, Clusterin, HTRA2, and Livin, while promoting the expression of cleaved Caspase-3. Supplementation with cIAP-1, HTRA2, or Livin partially reversed TIG3-induced Caspase-3 expression and cell death. **Conclusions:** Our findings suggest that TIG3 may contribute to the anti-melanoma effects of retinoic acid, with IAP family proteins playing a key role in the TIG3-mediated regulation of melanoma cell survival.

## 1. Introduction

Melanoma is a malignant tumor originating from melanocytes in the skin. Although it accounts for only about 1% of all skin cancers, it causes the majority of deaths related to skin cancer. According to GLOBOCAN 2020 data, there were approximately 325,000 new cases and about 57,000 deaths worldwide. By 2040, the numbers are projected to increase to 510,000 new cases and 96,000 deaths [1]. The incidence of melanoma shows marked geographic variation, with the highest rates observed in Australia and New Zealand, related to high ultraviolet exposure and ethnic characteristics. In contrast, the incidence rates are much lower in Asia (0.41 per 100,000) and Africa (0.9 per 100,000), primarily due to the darker skin phototypes and higher melanin content in these populations, which provide effective protection against ultraviolet radiation [2]. In the United States, approximately 104,960 new melanoma cases are expected to be diagnosed in 2025, including about 60,550 in men and 44,410 in women, with an estimated 8430 deaths. While its incidence is increasing, the related mortality rates have been in decline since 2013, resulting from advances in immunotherapies and targeted treatments (available online: https://www.cancer.org/cancer/types/melanoma-skin-cancer/about/key-statistics.html?utm_source=chatgpt.com; accessed on 4 June 2025).

Globally, the incidence and mortality rates of melanoma vary considerably. Once melanoma metastasizes, it becomes a life-threatening disease. Both BRAF inhibitors combined with mitogen-activated protein kinase (MAPK) inhibitors and immune checkpoint inhibitors have demonstrated significant clinical efficacy in metastatic melanoma, prolonging overall survival [3,4]. In clinical practice, these two therapeutic approaches are widely used and can be administered sequentially based on the patient’s genotype and disease condition. However, targeted therapies are prone to resistance, while immunotherapies are limited by high immune-related toxicities. Retinoic acid (RA), which is the active metabolite of vitamin A, has been widely studied for its potential in cancer therapy. The anti-cancer effects of RA have been demonstrated in various cancer models, including acute promyelocytic leukemia (APL), neuroblastoma, breast cancer, prostate cancer, lung cancer, and skin cancer. Notably, in the treatment of APL, RA promotes cancer cell differentiation and facilitates the degradation of the abnormal fusion protein PML-RARα, thereby restoring normal differentiation processes. In addition, RA has been shown to inhibit tumor cell proliferation, induce cell cycle arrest, and promote apoptosis—effects that are closely related to its regulation of signaling pathways such as Ras/MAPK and Wnt/β-catenin [5]. Large-scale studies and database analyses have shown that RA may effectively prevent the development of melanoma [6,7]. In melanoma research, RA has demonstrated multiple anti-tumor mechanisms, including the inhibition of tumor cell proliferation, the induction of cellular differentiation, the enhancement of chemotherapy efficacy, and the modulation of immune responses [8]. RA also influences the adhesion and migration of melanoma cells, potentially through the regulation of intercellular adhesion molecules such as ICAM-1, thereby suppressing tumor metastasis [9].

Tazarotene, which is a third-generation retinoid, regulates a group of target genes, including tazarotene-induced gene 1 (TIG1), TIG2, and TIG3, all of which participate in biological processes such as cell differentiation, proliferation, and apoptosis under RA control [10]. In recent years, the potential tumor-suppressive roles of these genes in melanoma have attracted growing interest. Studies have demonstrated that TIG1 inhibits proliferation and induces apoptosis in melanoma cells, likely by triggering endoplasmic reticulum stress and upregulating genes such as HERPUD1, BIP1, and DDIT3, leading to caspase-3 activation and subsequent cell death [11]. Furthermore, TIG1 has been shown to interact with VAC14, suppressing the mTOR signaling pathway and thereby limiting tumor cell growth [12]. TIG3 [13]—also known as retinoid-induced gene 1 (RIG1) [14], retinoic acid receptor responder 3 (RARRES3) [15], and phospholipase A and acyltransferase 4 (PLAAT4) [16]—is a retinoic acid-regulated gene that belongs to the PLAAT protein family (Phospholipase A/acyltransferase family) [17]. This gene is located on chromosome 11q12.1 in humans and encodes a small transmembrane protein that is involved in a range of physiological processes, including cellular differentiation, tumor suppression, and immune regulation [17,18,19].

TIG3 has been characterized as a tumor suppressor in skin cancers. It is robustly expressed in normal keratinocytes but is often downregulated in hyperproliferative skin lesions and skin tumors [20]. TIG3 is predominantly expressed in various epithelial tissues, especially in epidermal keratinocytes, where it plays a crucial role in cell differentiation and skin barrier formation [19,21]. Studies have shown that TIG3 possesses phospholipase or acyltransferase activity, which modulates the lipid composition of cellular membranes, thereby influencing intracellular signaling and cell fate decisions [22]. In several cancer types, TIG3 expression is significantly downregulated, suggesting its tumor-suppressing properties. TIG3 has been reported to inhibit cell cycle progression and promote apoptosis, particularly in models of squamous cell carcinoma, breast cancer, and prostate cancer [13,18,20,23,24,25]. These effects may be mediated, in part, through the modulation of the Ras signaling pathway [26,27,28]. Additionally, TIG3 has been implicated in interferon signaling and may play a role in innate immunity and antiviral responses [17,29], though this aspect remains under investigation. Despite sharing its name with RIG1, TIG3 is distinct from the well-known viral RNA sensor RIG-I (encoded by DDX58), with no structural or functional relationship between the two [30].

Although RA has been confirmed to suppress melanoma cell growth, existing studies predominantly focus on the function of its downstream effector—TIG1. In contrast, the role of TIG3 in melanoma remains poorly understood, despite its established tumor-suppressive functions in other skin malignancies. Therefore, this study aims to elucidate the anti-tumor mechanisms of TIG3 in melanoma cells. We employ RNA sequencing (RNA-Seq) and apoptosis protein arrays, using widely studied human melanoma cell lines A2058 and A375, which are commonly used for apoptosis research in melanoma. Through these approaches, we aim to systematically identify the downstream genes and signaling pathways regulated by TIG3. In doing so, we hope to uncover the specific intracellular signaling molecules through which TIG3 modulates melanoma cell growth and survival, ultimately providing a scientific basis for developing TIG3 as a potential therapeutic target for melanoma.

## 2. Materials and Methods

### 2.1. Gene Expression Analysis in Melanoma Tissues

A melanoma tissue cDNA array (MERT301) was obtained from OriGene Technologies (Rockville, MD, USA), comprising cDNAs from 3 normal skin tissues and 43 malignant melanoma samples at clinical stages III to IV. The primers used for TIG3 gene amplification were as follows: forward primer 5′-AAGTGAGTACCCCGGGGCTGG-3′ and reverse primer 5′-ACGGGCCGTGGTTGGTACTC-3′. Primers for β-actin were included in the commercial kit. Real-time PCR was performed under conditions described in a previous study [31], and TIG3 expression levels were normalized against β-actin expression.

### 2.2. Cell Culture and Gene Transfection

TIG3 and TIG3ΔC expression plasmids were prepared as previously described [26]. Expression vectors for cellular inhibitor of apoptosis proteins-1 (cIAP-1)-Flag (plasmid #27972), high temperature requirement A2 (HTRA2)-Flag (plasmid #15938), and EGFP-Survivin (plasmid #196908) were obtained from Addgene (Addgene, Watertown, MA, USA). Human melanoma cell lines A2058 and A375, which are commonly used for studying melanoma cell growth and death [32], were purchased from the Bioresource Collection and Research Center (BCRC, Hsinchu, Taiwan). The cells were cultured and subcultured following the manufacturer’s protocols. Gene transfections were performed using Lipofectamine 2000 reagent (Invitrogen, Carlsbad, CA, USA), as described in previous studies [11,12]. Routine quality control included DAPI staining and PCR-based detection to eliminate potential mycoplasma contamination.

### 2.3. Cell Viability and Cell Death Assays

Cell viability and death were assessed as described in prior studies [11,16]. Briefly, A2058 or A375 cells (2 × 10^4^ cells per well) were seeded in triplicate in 24-well plates, before being incubated for 24 h at 37 °C in media containing 10% fetal bovine serum (FBS; Gibco, Thermo Fisher Scientific, Waltham, MA, USA). After transfection with either an empty vector, the TIG3-myc-his expression vector, or the TIG3ΔC-myc-his expression vector—or co-transfection with TIG3-myc-his and cIAP-1-Flag, HTRA2-Flag, or EGFP-Survivin expression vectors—the media were replaced after 6 h with fresh 10% FBS-containing media, before being incubated overnight. The next day, the media were replaced with 1% FBS-containing media, and cells were cultured for an additional 24–48 h.

Cell death was evaluated using the Cytotoxicity Detection Kit (LDH Assay, Roche Diagnostics, Mannheim, Germany) according to the manufacturer’s protocol. This assay measures the release of lactate dehydrogenase (LDH) into the culture medium from damaged cells, which reflects the loss of membrane integrity. The absorbance of the reaction product was measured at 490 nm. Cell viability was assessed using the WST-1 reagent (Roche Diagnostics, Mannheim, Germany). This colorimetric assay detects mitochondrial dehydrogenase activity, which reduces the WST-1 reagent to a soluble formazan dye. The amount of formazan produced correlates with the number of metabolically active cells and was measured at 450 nm. All absorbance values were quantified using a microplate reader (Infinite F200, Tecan, Durham, NC, USA).

### 2.4. RNA-Seq Analysis

RNA-seq analysis was performed as previously reported [11]. In brief, A2058 cells were transfected with either an empty vector, the TIG3-myc-his expression vector, or the TIG3ΔC-myc-his expression vector; after 24 h, they were harvested using TRIZOL reagent (Invitrogen, Carlsbad, CA, USA). RNA extraction and sequencing were carried out at the Biotools Microbiome Research Center (Taipei, Taiwan). Raw reads were quality-filtered using Trimmomatic to generate clean reads, which were then aligned to the reference genome using HISAT2. Gene expression levels (raw read counts) were calculated using featureCounts and were normalized using the RLE, TMM, or FPKM method. Differential gene expression was identified using DESeq2 with selection criteria of |Fold Change| > 2 and adjusted *p*-value < 0.05 based on biological replicates.

### 2.5. Apoptosis Array Analysis

The Proteome Profiler™ Human Apoptosis Array Kit (ARY009, R&D Systems, Minneapolis, MN, USA), which is capable of detecting 35 apoptosis-related proteins, was used to evaluate the involvement of TIG3 in apoptosis signaling in A2058 cells. After 24 h of transfection with either an empty vector, the TIG3-myc-his expression vector, or the TIG3ΔC-myc-his expression vector, cell lysates were prepared using the kit’s lysis buffer 17. Protein concentrations were determined using the Bio-Rad Protein Assay (Bio-Rad Laboratories, Hercules, CA, USA). A total of 300 μg of protein was loaded per array, incubated overnight, and subsequently treated with detection antibodies for 1 h and Streptavidin-horseradish peroxidase (HRP) for 30 min. Signal development was performed via chemiluminescence and was detected using an e-blot Touch Imager (Pudong, Shanghai, China).

### 2.6. Western Blot Analysis

Western blot was conducted as previously described [12]. A2058 and A375 cells (1 × 10^6^) were seeded in 10 cm culture dishes and were transfected with either an empty vector, the TIG3-myc-his expression vector, or the TIG3ΔC-myc-his expression vector, or were co-transfected with TIG3-myc-his and cIAP-1-Flag, HTRA2-Flag, or EGFP-Survivin expression vectors, before being incubated for 24 h. Cell lysates were prepared for protein analysis. The primary antibodies used were as follows: anti-cIAP-1, anti-HTRA2, anti-Livin, anti-total Caspase-3, and anti-cleaved Caspase-3 (Asp175) (all from Cell Signaling Technology, Beverly, MA, USA; dilution 1:1000), and anti-β-Actin antibody (Sigma-Aldrich, St. Louis, MO, USA; dilution 1:5000). The secondary antibodies were HRP-conjugated goat anti-mouse or anti-rabbit IgG (Calbiochem, Darmstadt, Germany; dilution 1:5000).

### 2.7. Flow Cytometry Analysis of Cell Cycle Distribution

A2058 and A375 cells (1 × 10^6^) were seeded in 10 cm dishes. After 24 h of transfection with either an empty vector, the TIG3-myc-his expression vector, or the TIG3ΔC-myc-his expression vector, or co-transfection with TIG3-myc-his and cIAP-1-Flag, HTRA2-Flag, or EGFP-Survivin expression vectors, cells were harvested and fixed in cold 80% ethanol at −20 °C for at least 12 h. The fixed cells were stained with propidium iodide solution (20 μg/mL propidium iodide, 0.1% Triton X-100, and 0.2 mg/mL RNase A) for 30 min and were analyzed using a CytoFLEX S flow cytometer (Beckman Coulter, IN, USA). Cell cycle profiles and sub-G1 populations were quantified using the instrument’s built-in software (CyExpert version 2.5).

### 2.8. Statistical Analysis

All data were derived from at least three independent experiments and are presented as mean ± standard deviation. Statistical comparisons were performed using one-way ANOVA followed by Dunnett’s *t*-test. A *p*-value < 0.05 was considered statistically significant.

## 3. Results

### 3.1. TIG3 Expression Inhibits Cell Growth and Promotes Cell Death in Melanoma Cells

**Figure 1:**
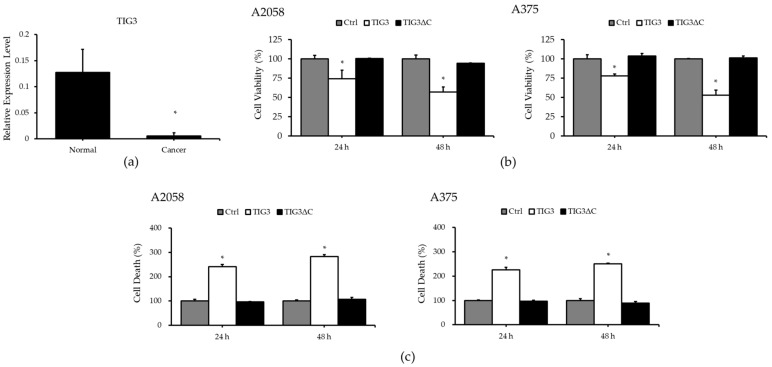
Effects of TIG3 on cell viability and death in A2058 and A375 cells. TIG3 mRNA expression in malignant melanoma tissues was analyzed using a commercial melanoma cDNA tissue array (MERT301) via real-time PCR (**a**). A2058 or A375 cells were transfected with either an empty vector, the TIG3-myc-his expression vector, or the TIG3ΔC-myc-his expression vector, before being incubated for 24–48 h. Cell viability (**b**) and cell death (**c**) were assessed using WST-1 assays and LDH release assays, respectively (*n* = 3). * *p* < 0.05 compared with the control group.

To investigate the potential function of TIG3 in melanoma, we first analyzed TIG3 expression in normal skin tissue and melanoma samples using a melanoma tissue array. As shown in Figure 1a, TIG3 was highly expressed in normal skin tissues but was significantly downregulated in melanoma tissues. In addition, TIG3 expression was barely detectable in human melanoma cell lines A2058 and A375. Treatment with all-trans RA (ATRA) was able to induce TIG3 expression in A375 cells (Supplementary Figure S1). To further explore the role of TIG3 in melanoma cells, we overexpressed TIG3 in A2058 and A375 cells, assessing its effects on cell viability and cell death. The results from Figure 1b,c indicate that TIG3 expression in A2058 cells for 24–48 h led to a decrease in cell viability and a marked increase in cell death.

Previous studies have shown that TIG3′s cellular functions depend on its membrane localization, while a loss of this ability abolishes its activity [20,23,26,33]. To evaluate whether this property is required for TIG3′s function in melanoma cells, we expressed a membrane localization-deficient mutant—TIG3ΔC—in A2058 cells. The results demonstrated that TIG3ΔC had no significant effect on cell viability or cell death in A2058 cells (Figure 1b,c). Similar findings were observed in A375 cells, where TIG3 suppressed cell viability and promoted cell death, while TIG3ΔC showed no noticeable effect (Figure 1b,c).

### 3.2. Identification of TIG3-Regulated Targets Using RNA Sequencing and Protein Array Analyses

To further investigate the targets through which TIG3 suppresses melanoma cell viability and induces cell death, we analyzed the downstream genes that are regulated by TIG3, as well as the expression of apoptosis-related proteins. Although TIG1 is not a transcription factor, it has been shown to activate downstream gene expression indirectly through interactions with other regulatory molecules [11,34]. Therefore, we could not exclude the possibility that TIG3, despite lacking intrinsic transcription factor activity, might exert a similar effect by indirectly activating downstream genes via intermediate signaling components.

To address this, we first conducted RNA-Seq to examine the changes in gene expression following TIG3 overexpression in the A2058 melanoma cell line. The results from the volcano plot and MA plot revealed that compared to the control group, only the expression of the TIG3 gene itself was significantly affected upon TIG3 expression (Supplementary Figure S2a,b). The heatmap analysis further confirmed that only TIG3 expression was notably altered (Supplementary Figure S2c). In addition, we observed no significant differences between the gene regulatory effects of TIG3 and its C-terminal deletion mutant (TIG3ΔC) in A2058 cells (Supplementary Figure S2). These findings suggest that the mechanism by which TIG3 alters cell viability and promotes cell death in A2058 melanoma cells is not mediated through the direct or indirect transcriptional regulation of downstream genes.

**Figure 2:**
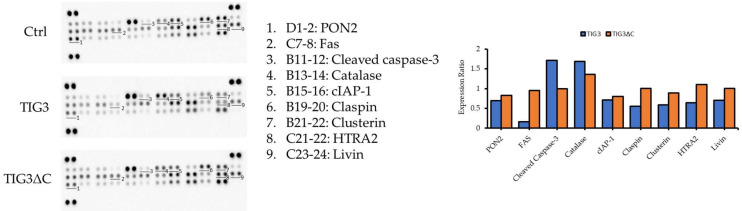
Effects of TIG3 on the expression of apoptosis-related proteins. A2058 cells were transfected with either an empty vector, the TIG3-myc-his expression vector, or the TIG3ΔC-myc-his expression vector for 24 h. Cell lysates were collected and analyzed using a Human Apoptosis Array Kit to detect protein expression levels. Bar graphs present the quantitative results of the target protein. The expression level of the target protein was first normalized to its corresponding reference spots, which served as internal controls. Subsequently, these values were further normalized to the expression level of the same target protein in the control group to obtain relative expression levels.

Subsequently, we used a protein array to assess the expression of apoptosis-related proteins in A2058 cells following TIG3 expression. As shown in Figure 2, TIG3 upregulated Catalase and cleaved Caspase-3, while downregulated PON2, Fas, cIAP-1, Claspin, Clusterin, HTRA2, and Livin. Interestingly, TIG3ΔC, like TIG3, was able to suppress PON2 and enhance Catalase expression (Figure 2). Based on the available expression vectors and validated antibodies, we selected cIAP-1, HTRA2, and Livin for further analysis.

### 3.3. Analysis of TIG3′s Effects on Apoptosis-Related Protein Expression

**Figure 3:**
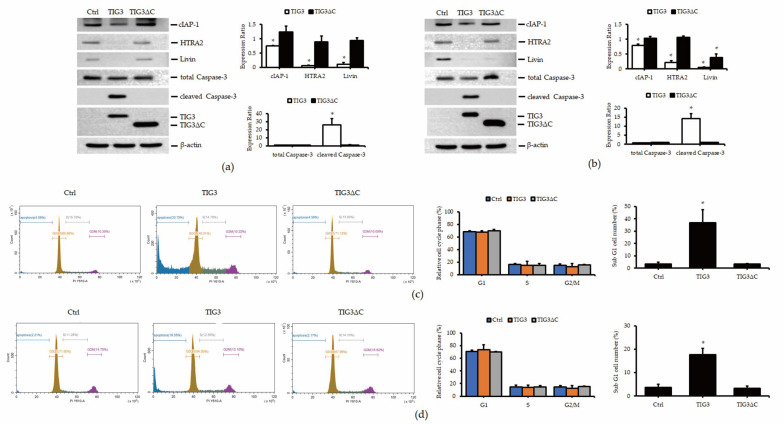
TIG3-induced expression changes in apoptosis-related proteins in A2058 and A375 cells. A2058 (**a**) or A375 (**b**) cells were transfected with either an empty vector, the TIG3-myc-his expression vector, or the TIG3ΔC-myc-his expression vector for 24 h. The expressions of cIAP-1, HTRA2, Livin, and cleaved Caspase-3 were analyzed via Western blot. Bar graphs present the quantitative analysis of target proteins obtained from three independent Western blot experiments. For each sample, the expression level of the target protein was first normalized to the corresponding β-actin level, serving as an internal loading control. Subsequently, the normalized values were further normalized to the expression level of the same target protein in the control group to determine the relative expression levels. Alternatively, A2058 (**c**) or A375 (**d**) cells were stained with propidium iodide and analyzed using flow cytometry (*n* = 3). * *p* < 0.05 compared with the control group.

To validate the protein array results, we performed Western blot to examine the effects of TIG3 and TIG3ΔC on the expression of key apoptosis-related proteins. As shown in Figure 3a, TIG3 (but not TIG3ΔC) significantly suppressed the expression of cIAP-1, HTRA2, and Livin in A2058 cells, while also inducing cleaved Caspase-3. Similar results were observed in A375 cells, where TIG3 inhibited cIAP-1, HTRA2, and Livin, as well as promoted the expression of cleaved Caspase-3 (Figure 3b).

Both cIAP-1 and Livin are members of the intracellular inhibitor of apoptosis proteins (IAPs) family [35], and HTRA2 is known to modulate IAP activity [36]. These proteins can influence apoptosis by regulating the Caspase cascade. We further examined whether TIG3 affects cell cycle progression and DNA fragmentation. The flow cytometry results (Figure 3c) showed that TIG3 did not significantly alter the cell cycle in A2058 cells but did increase the proportion of sub-G1 cell populations, which is indicative of apoptosis. A similar increase in sub-G1 cells was observed in A375 cells (Figure 3d).

### 3.4. Impact of Apoptosis-Related Proteins on TIG3-Induced Caspase-3 Activation

**Figure 4:**
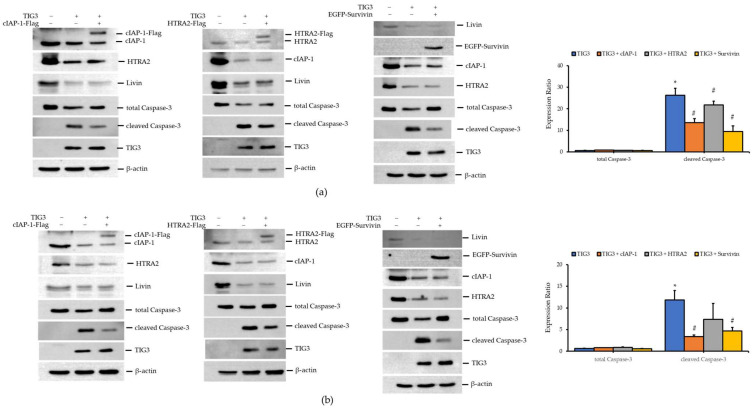
Effects of cIAP-1, HTRA2, and Survivin on TIG3-induced Caspase-3 expression in A2058 and A375 cells. A2058 (**a**) or A375 (**b**) cells were transfected with either an empty vector, the TIG3-myc-his expression vector, or were co-transfected with TIG3-myc-his and cIAP-1-Flag, HTRA2-Flag, or EGFP-Survivin expression vectors. After 24 h, cell lysates were collected and the expression levels of cIAP-1, HTRA2, Livin, and Caspase-3 were analyzed via Western blot. Bar graphs present the quantitative analysis of target proteins obtained from three independent Western blot experiments. For each sample, the expression level of the target protein was first normalized to the corresponding β-actin level, serving as an internal loading control. Subsequently, the normalized values were further normalized to the expression level of the same target protein in the control group to determine the relative expression levels. * *p* < 0.05 compared with the control group. # *p* < 0.05 compared with the TIG3-expressing group.

Given that TIG3 suppresses cIAP-1, HTRA2, and Livin expression while inducing cleaved Caspase-3 and promoting cell death, we next explored whether the re-expression of these proteins could reverse TIG3′s effects. Due to the unavailability of a Livin expression vector, we substituted it with Survivin, which is a functionally similar IAP family member [37,38,39]. As shown in Figure 4a, the expression of cIAP-1 in A2058 cells did not affect the TIG3-mediated suppression of HTRA2 and Livin. Similarly, the expression of HTRA2 or Survivin did not alter TIG3′s effect on other apoptosis-related proteins. The same trend was observed in A375 cells (Figure 4b), suggesting that TIG3 regulates cIAP-1, HTRA2, and Livin expression via independent pathways.

We then assessed whether the expression of these apoptosis regulators affected the TIG3-induced activation of Caspase-3. In A2058 cells, the co-expression of TIG3 with cIAP-1, HTRA2, or Survivin attenuated the upregulation of cleaved Caspase-3 induced by TIG3 (Figure 4a). Similar effects were observed in A375 cells; co-expression of TIG3 with cIAP-1 or Survivin expression (Figure 4b).

### 3.5. Effects of Apoptosis-Related Proteins on TIG3-Mediated Growth Inhibition and Cell Death

**Figure 5:**
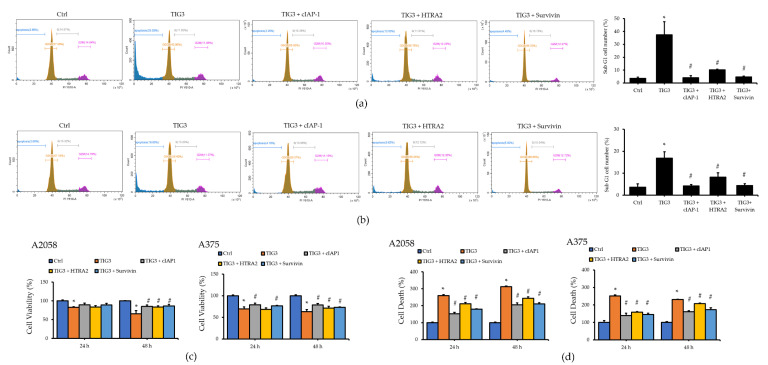
Effects of cIAP-1, HTRA2, and Survivin on TIG3-mediated cell viability and death in A2058 and A375 cells. A2058 (**a**) or A375 (**b**) cells were transfected with either an empty vector, the TIG3-myc-his expression vector, or were co-transfected with TIG3-myc-his and cIAP-1-Flag, HTRA2-Flag, or EGFP-Survivin expression vectors. After 24 h, cells were stained with propidium iodide and analyzed using flow cytometry. Alternatively, after 24–48 h of expression, cell viability (**c**) and cell death (**d**) were assessed using WST-1 and LDH release assays (*n* = 3). * *p* < 0.05 compared with the control group. # *p* < 0.05 compared with the TIG3-expressing group.

Since cIAP-1, HTRA2, and Survivin suppressed TIG3-induced Caspase-3 activation, we examined whether they also affect the TIG3-induced accumulation of sub-G1 cells. As shown in Figure 5a, the expression of cIAP-1, HTRA2, or Survivin in A2058 cells significantly reduced the TIG3-induced increase in sub-G1 cell populations. Notably, the co-expression of TIG3 with either cIAP-1 or Survivin restored the sub-G1 population to baseline levels. A similar reduction was observed in A375 cells’ co-expression of TIG3 with either cIAP-1 or Survivin (Figure 5b).

We further evaluated whether these proteins could reverse the TIG3-induced changes in cell viability and death. As illustrated in Figure 5c,d, the expression of cIAP-1, HTRA2, or Survivin partially rescued the TIG3-induced decreases in cell viability and the increases in cell death in both A2058 and A375 cells. These results indicate that while cIAP-1 and Survivin can attenuate the increase in sub-G1 cells, they cannot fully reverse TIG3-induced cell death (Figure 5a,c,d). This suggests that TIG3-mediated cell death may occur through both Caspase-dependent pathways regulated by cIAP-1 and Survivin, as well as through other (yet to be identified) mechanisms. Furthermore, to determine whether oxidative stress plays a role in TIG3-mediated cell death, we investigated whether TIG3 affects the production of reactive oxygen species (ROS) in A2058 cells. As shown in Supplementary Figure S3, the expression of TIG3 alone or in combination with cIAP-1, Survivin, or HTRA2 did not alter intracellular ROS levels. These results suggest that TIG3 does not regulate cell death through the oxidative stress pathway.

## 4. Discussion

In this study, although TIG3 did not significantly downregulate the mRNA expression of any inhibitor of apoptosis protein (IAP) family genes based on RNA sequencing analysis, we observed a clear decrease in the protein levels of several IAP members, such as cIAP-1 and Livin, in the apoptosis protein array. This discrepancy suggests that TIG3 may regulate IAPs via post-transcriptional or post-translational, rather than transcriptional mechanisms. Previous studies have shown that IAP expression can be modulated by post-transcriptional mechanisms such as microRNAs and RNA stability [40]. In addition, IAP stability is known to be regulated by the ubiquitin–proteasome system. For instance, cIAP-1 undergoes rapid degradation through self-ubiquitination, and its stability can be influenced by external stimuli [41]. Therefore, we speculate that TIG3 may reduce IAP levels by decreasing their stability, enhancing their degradation, or interfering with microRNA-mediated regulation, rather than directly suppressing IAP gene transcription. These findings suggest that TIG3 may influence cell fate decisions by regulating IAPs through non-canonical pathways during apoptosis, highlighting the need for further investigation into its roles in protein modification and protein–protein interactions.

Although studies have suggested that RA can effectively prevent melanoma formation, its current application is limited to dietary supplementation due to adverse effects that hinder its widespread therapeutic use. To address this, several generations of retinoic acid derivatives have been developed with the goal of maintaining therapeutic efficacy while minimizing side effects. Among these, third-generation derivative tazarotene has seen the most widespread use [10,42]. Tazarotene is currently approved for topical use and has been evaluated in several clinical trials as an adjuvant therapy in combination with the immunomodulatory agent Imiquimod for treating cutaneous melanoma [43], suggesting its therapeutic potential. The mechanism of action of tazarotene has been extensively studied. Beyond acting as an antagonist of IFN-γ or AP1 receptors, its biological activity is closely associated with its induced genes, particularly TIG1 and TIG3. TIG1 has been well documented in the context of melanoma [11,12,44,45]. In this study, we found that TIG3, another gene regulated by tazarotene, suppresses melanoma cell activity and promotes cancer cell death. TIG3 downregulates the expression of apoptosis-inhibiting proteins such as cIAP-1 and Livin, thereby activating the caspase-3 pathway and inducing apoptosis. Although there are currently no known drugs other than retinoids that can directly induce TIG3 expression, our findings support the feasibility of developing novel therapeutic strategies targeting the TIG3 pathway. This study also provides a potential direction for molecular targeted therapy in melanoma.

IAPs are a family of proteins that can inhibit apoptosis either directly or indirectly. Major members include XIAP (X-linked IAP), cIAP1, cIAP2, Livin, and Survivin. These proteins are involved in regulating cell survival, immune responses, inflammation, and the development and drug resistance of tumors [35,41]. In cancer cells, IAPs are often overexpressed. By inhibiting caspases—especially Caspase-3, -7, and -9—they block both intrinsic and extrinsic apoptotic signaling pathways, allowing tumor cells to evade immune surveillance and apoptosis, thereby promoting tumorigenesis and metastasis [46,47]. For example, Livin directly binds and inhibits activated Caspase-3 and -9, playing a critical role in various tumors such as melanoma, lung cancer, and neuroblastoma [48,49,50]. cIAP-1, on the other hand, modulates signaling pathways like NF-κB and MAPK through ubiquitination, enhancing cell survival and inflammatory responses [51,52]. The overexpression of IAPs is also strongly associated with poor prognosis in cancers such as liver, gastric, breast, and brain tumors [46,53,54,55,56]. Therefore, IAPs have emerged as important targets in cancer drug development. Various IAP inhibitors are currently in preclinical or clinical development, with Smac mimetics being the most prominent. These agents mimic endogenous IAP antagonists (e.g., Smac/DIABLO), bind to IAPs, promote their auto-degradation, and relieve their inhibition on caspases, thereby triggering apoptosis [57,58]. Overall, IAP family proteins play a central role in cancer cell survival and treatment resistance. Targeting them not only induces cancer cell apoptosis but may also help reshape the tumor immune microenvironment, paving the way for more effective targeted cancer therapies.

HTRA2 (also known as Omi) is a serine protease located in the mitochondrial intermembrane space; it is a member of the HtrA family. Upon cellular stress or apoptotic stimuli, HTRA2 is released into the cytosol, where it plays a key role in regulating apoptosis [59]. HTRA2 exhibits dual roles in cancer development—while it promotes apoptosis and has tumor-suppressive potential, its dysregulation or reduced expression is also associated with tumor cell survival and resistance [60]. The pro-apoptotic function of HTRA2 relies on both its serine protease activity and an N-terminal Reaper-like IAP-binding motif (IBM), which allows HTRA2 to bind to IAPs such as XIAP, cIAP1, and cIAP2. This disrupts the interaction between IAPs and caspases, lifting their inhibition on caspase-3 and caspase-9, thus promoting apoptosis [36]. Moreover, HTRA2′s protease activity can directly degrade IAPs, further enhancing apoptotic signaling [36]. Research has shown that HTRA2 expression is significantly downregulated in multiple cancers, including gastric, liver, and prostate cancers, a trend that inversely correlates with IAP overexpression and may represent a mechanism for tumor cell survival [61]. Thus, HTRA2 plays a critical role in maintaining apoptotic signaling balance and tumor suppression. Its antagonistic relationship with IAPs provides insight into the mechanisms of apoptosis evasion in cancer, offering a potential avenue for therapeutic intervention.

The observation that IAP can reverse the effects induced by TIG3 suggests that TIG3 may inhibit tumor cell survival not only through apoptosis, but also by engaging other cell death or survival pathways, such as autophagy or necroptosis. Autophagy is an intracellular degradation and recycling process typically activated under stress or nutrient deprivation. While it generally serves a protective role, excessive autophagy can also lead to cell death. Previous studies have shown that TIG1, another RA-induced gene, may promote autophagy activation by modulating the mTOR or AMPK signaling pathways, thereby influencing cell fate [12,62]. Whether TIG3 shares similar functionality remains unexplored. In addition, necroptosis—a form of programmed necrosis mediated by the RIPK1/RIPK3/MLKL axis [63]—might also be involved in TIG3′s mechanism of action. If IAP inhibit necroptosis by suppressing RIPK1 activity, this could explain their ability to interfere with TIG3-mediated effects. Therefore, future studies incorporating expression analyses of key markers such as LC3B, p62, and p-MLKL may help elucidate TIG3′s potential role in regulating these non-canonical cell death pathways, ultimately enhancing our understanding of its anti-tumor mechanisms.

The above introduction of HTRA2 suggests that its expression can suppress IAP activity, leading to the activation of the caspase cascade, as well as the induction of apoptosis. Consequently, HTRA2 is often found to be downregulated during cancer development. However, while we observed that TIG3 downregulates the expression of IAP family members, we also found that it suppresses HTRA2 expression. Furthermore, the co-expression of HTRA2 in TIG3-expressing melanoma cells counteracts TIG3-induced apoptosis. This contradicts prior studies that emphasize HTRA2′s pro-apoptotic role. We hypothesize that in cells that are already deficient in IAPs due to TIG3 expression, HTRA2 may exert different, possibly compensatory, functions. Similarly, research by Kang et al. showed that a loss of HTRA2 activity causes pronounced physiological abnormalities. In HtrA2 knockout mice, early aging features, such as organ atrophy, functional decline, increased cellular damage, and elevated oxidative stress, and apoptotic signaling were observed in multiple non-neuronal tissues (e.g., liver, lung, and kidney) [64]. These findings suggest that HTRA2 is essential for maintaining tissue homeostasis and mitigating oxidative damage and aging. Therefore, the downregulation of HTRA2 by TIG3 may also contribute to the loss of homeostasis and the induction of apoptosis. Although HTRA2 expression is known to be associated with intracellular oxidative stress, our study showed that TIG3, unlike TIG1, does not enhance the expression of oxidative stress-related proteins [11], nor does it alter intracellular ROS levels. Therefore, we speculate that after TIG3 causes a decrease in HTRA2 expression, the subsequent reduction in intracellular HTRA2 levels may induce apoptosis not through oxidative stress pathways, but rather via other cellular homeostasis mechanisms such as ion balance, lipid and membrane stability, or autophagy and cellular clearance processes. Following TIG3′s regulation of HTRA2 expression, the detailed mechanisms by which changes in HTRA2 levels lead to cell death still require further experimental validation.

In our study, the exogenous expression of cIAP-1, HTRA2, or Survivin reduced TIG3-induced caspase activation and even completely diminished the sub-G1 cell population. However, these proteins did not fully restore cell viability or prevent TIG3-induced cell death in melanoma cells. Similarly, the inhibition of caspase-3 did not entirely block TIG3-induced cell death [10], suggesting that TIG3 may also activate caspase-independent cell death pathways. Our experimental approach primarily focuses on gene and protein expressions to assess the activity of gene products. Given that TIG3 localizes primarily to the endoplasmic reticulum and Golgi apparatus and has the ability to regulate the lipid composition of the cell membrane [22], it is possible that TIG3 regulates cellular homeostasis through these lipid-modifying activities. However, such changes are currently beyond the scope of our research. Whether lipid modification–dependent proteins or other targets are involved in TIG3-mediated regulation of melanoma cell growth remains unknown. To fully understand how TIG3 maintains cellular homeostasis and controls melanoma progression, the development of assays capable of detecting the activity of lipid-modified proteins is required.

## 5. Conclusions

In this study, we found that the RA-induced gene TIG3 is highly expressed in normal skin tissues but is significantly downregulated in malignant melanoma tissues, suggesting a potential tumor-suppressive role of TIG3 in melanoma development. Although TIG3 expression in melanoma cells did not alter the transcriptional regulation of other genes, protein expression profiling and Western blot analyses revealed that TIG3 downregulates members of the IAP family, including cIAP-1 and Livin, thereby activating Caspase-3 and inducing apoptosis. Furthermore, re-expression of these TIG3-suppressed IAP in melanoma cells partially rescued the apoptotic effects caused by TIG3. These findings collectively suggest that TIG3 may regulate melanoma cell survival through modulation of IAP family proteins and may serve as a potential therapeutic target for melanoma treatment.

## Data Availability

All data generated or analyzed during this study are included in the article; further enquiries can be directed to the corresponding author.

## Supplementary Materials

The following supporting information can be downloaded at https://www.mdpi.com/article/10.3390/biomedicines13071749/s1. Figure S1: Expression of TIG3 in A2058 and A375 cells; Figure S2: RNA-seq analysis of genes differentially regulated by TIG3 in A2058 cells; Figure S3: Effects of TIG3 on ROS generation in A2058 cells.

## Abbreviations

The following abbreviations are used in this manuscript:

MAPK	Mitogen-activated protein kinase
RA	Retinoic acid
APL	Acute promyelocytic leukemia
TIG1	Tazarotene-induced gene 1
TIG3	Tazarotene-induced gene 3
RIG1	Retinoid-induced gene 1
RARRES3	Retinoic acid receptor responder 3
PLAAT4	Phospholipase A and acyltransferase 4
RNA-Seq	RNA sequencing
cIAP-1	Cellular inhibitor of apoptosis proteins-1
HTRA2	High temperature requirement A2
LDH	Lactate dehydrogenase
IAP	Inhibitors of Apoptosis Protein
