# Supplementary material for: Tazarotene-Induced Gene 3 (TIG3) Induces Apoptosis in Melanoma Cells Through the Modulation of Inhibitors of Apoptosis Proteins
Source: Biomedicines. 2025 Jul 17;13(7):1749. doi: 10.3390/biomedicines13071749 (PMC12292944; doi:10.3390/biomedicines13071749)
Supplement: Supplementary file 1 [file biomedicines-13-01749-s001.zip › biomedicines-3718897-supplementary.pdf]

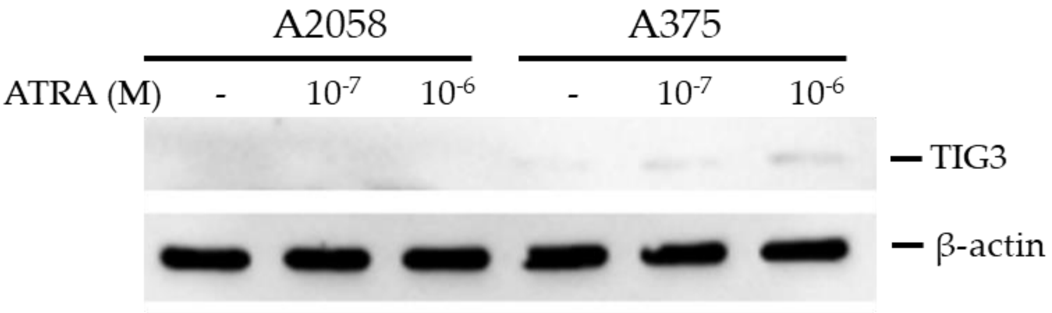

Supplementary Figure S1. Expression of TIG3 in A2058 and A375 cells. A2058 or A375 cells plated in 6-well dishes were treated daily with the indicated concentration of all-trans-retinoic acid (ATRA) for 48 h. Cell lysates were collected and the expression levels of TIG3 were analyzed via Western blot.

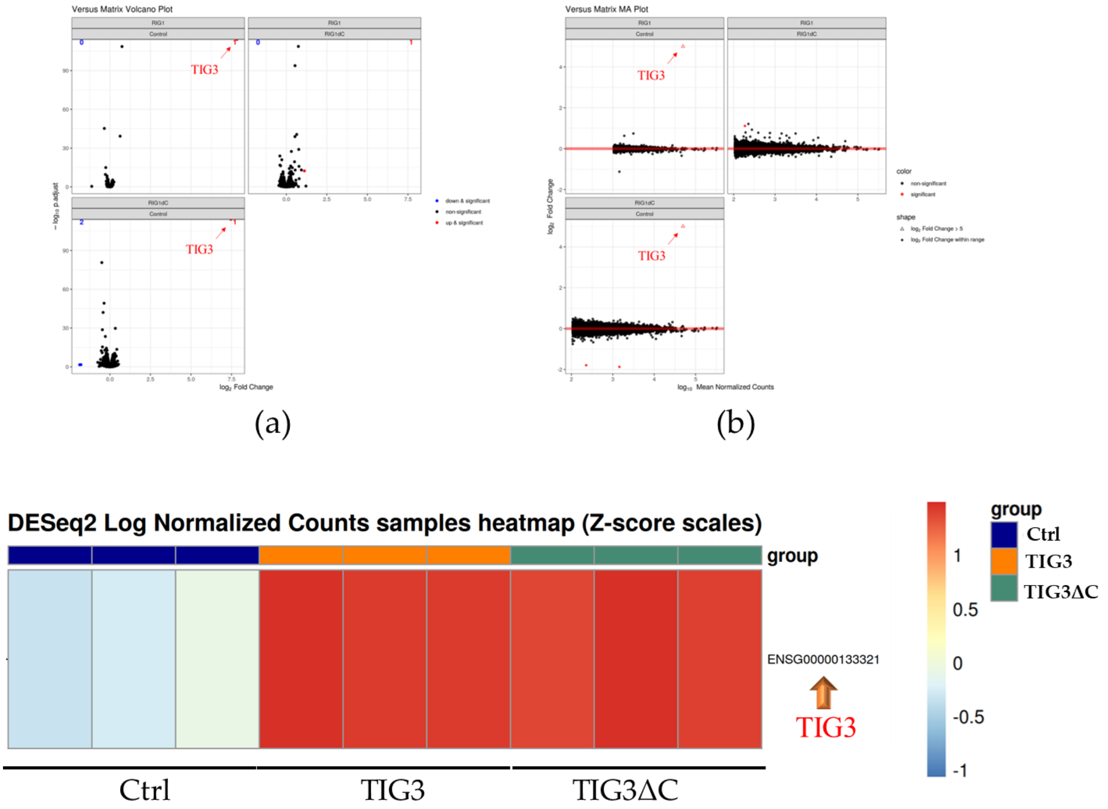

(c)

Supplementary Figure S2. RNA-seq analysis of genes differentially regulated by TIG3 in A2058 cells. A2058 cells were transfected with either an empty vector, the TIG3-myc-his expression vector, or the TIG3ΔC-myc-his expression vector, before being incubated for 24 h. RNA-seq was performed to analyze the gene expression profiles. Differentially expressed genes are displayed in a volcano plot (a) and an MA plot (b), with red dots indicating statistically significant differences. A heatmap (c) shows the expression levels of genes among the groups.

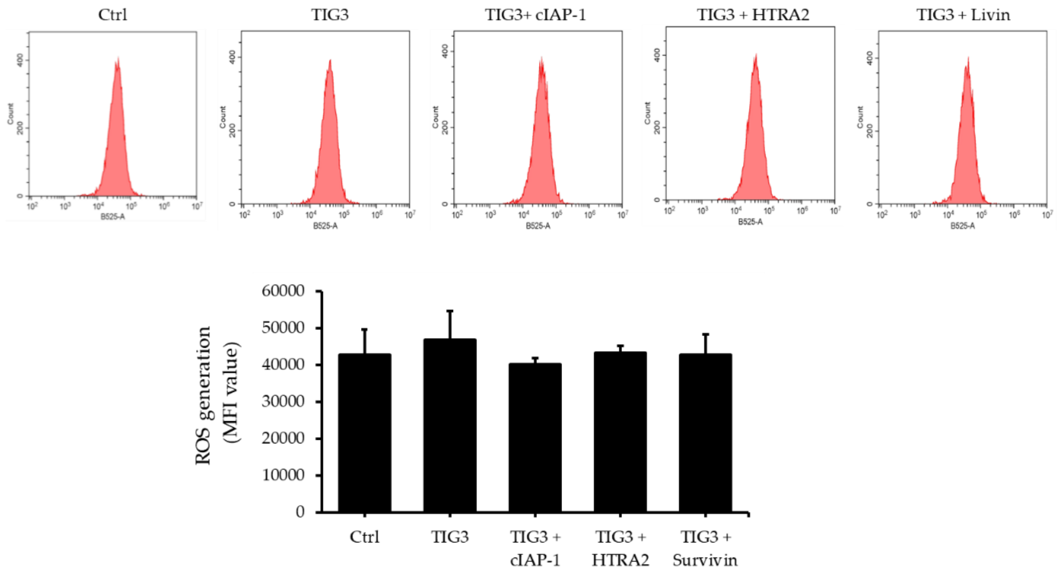

Supplementary Figure S3. Effects of TIG3 on ROS generation in A2058 cells. A2058 cells were transfected with either an empty vector, the TIG3-myc-his expression vector, or were co-transfected with TIG3-myc-his and cIAP-1-Flag, HTRA2-Flag, or EGFP-Survivin expression vectors. After 24 h, cells were stained with Dihydrorhodamine 123 (DHR123) dye and analyzed for ROS generation using flow cytometry (n=3).
